# Potential molecular link between the β-amyloid precursor protein (APP) and hypoxanthine-guanine phosphoribosyltransferase (HGprt) enzyme in Lesch-Nyhan disease and cancer

**DOI:** 10.3934/Neuroscience.2021030

**Published:** 2021-10-28

**Authors:** Khue Vu Nguyen

**Affiliations:** Former Institution Attended:; 1 Department of Medicine, Biochemical Genetics and Metabolism, The Mitochondrial and Metabolic Disease Center, School of Medicine, University of California, San Diego, Building CTF, Room C-103, 214 Dickinson Street, San Diego, CA 92103-8467, USA; 2 Department of Pediatrics, University of California, San Diego, School of Medicine, San Diego, La Jolla, CA 92093-0830, USA

**Keywords:** β-amyloid precursor protein (APP), hypoxanthine-guanine phosphoribosyltransferase (HGprt) enzyme, *APP* gene, hypoxanthine phosphoribosyltransferase 1 (*HPRT1*) gene, epistasis, epigenetics, alternative splicing, Lesch-Nyhan disease, COVID-19, thrombosis, cancer, antisense drugs

## Abstract

Lesch-Nyhan disease (LND) is a rare X-linked inherited neurogenetic disorders of purine metabolic in which the cytoplasmic enzyme, hypoxanthine-guanine phosphoribosyltransferase (HGprt) is defective. Despite having been characterized over 60 years ago, however, up to now, there is no satisfactory explanation of how deficits in enzyme HGprt can lead to LND with the development of the persistent and severe self-injurious behavior. Recently, a role for epistasis between the mutated hypoxanthine phosphoribosyltransferase 1 (*HPRT1*) and the β-amyloid precursor protein (APP) genes affecting the regulation of alternative APP pre-mRNA splicing in LND has been demonstrated. Furthermore, there were also some reported cases of LND developing thrombosis while APP is an important regulator of vein thrombosis and controls coagulation. Otherwise, the surface expression of HGprt enzyme was also observed in several somatic tissue cancers while APP and the APP-like protein-2 (APLP2) are deregulated in cancer cells and linked to increased tumor cell proliferation, migration, and invasion. The present review provides a discussion about these findings and suggests a potential molecular link between APP and HGprt via epistasis between *HPRT1* and *APP* genes affecting the regulation of alternative APP pre-mRNA splicing. As a perspective, expression vectors for HGprt enzyme and APP are constructed as described in Ref. # 24 (Nguyen KV, Naviaux RK, Nyhan WL (2020) Lesch-Nyhan disease: I. Construction of expression vectors for hypoxanthine-guanine phosphoribosyltransferase (HGprt) enzyme and amyloid precursor protein (APP). *Nucleosides Nucleotides Nucleic Acids* 39: 905–922), and they could be used as tools for clarification of these issues. In addition, these expression vectors, especially the one with the glycosyl-phosphatidylinositol (GPI) anchor can be used as a model for the construction of expression vectors for any protein targeting to the cell plasma membrane for studying intermolecular interactions and could be therefore useful in the vaccines as well as antiviral drugs development (studying intermolecular interactions between the spike glycoprotein of the severe acute respiratory syndrome coronavirus 2, SARS-CoV-2, as well as its variants and the angiotensin-converting enzyme 2, ACE2, in coronavirus disease 2019 (COVID-19) [43],[44], for example).

## 1. Introduction

Lesch-Nyhan disease (LND) is a rare X-linked inherited neurogenetic disorder of purine metabolism affecting 1 in 380,000 people, and caused by deficiency of the soluble cytoplasmic hypoxanthine-guanine phosphoribosyltransferase (HGprt) enzyme (EC. 2.4.2.8; MIM 300800). This enzyme plays a central role in the generation of purine nucleotides from degraded DNA through the purine salvage pathways [1],[2]. LND is characterized by hyperuricemia, gout, nephrolithiasis, tophi, motor disability, intellectual impairment, and self-injurious behaviors such as self-biting, self-hitting, eye poking, and others. Complete or severe deficiency of HGprt activity leads to LND (MIM 300322). Self-injurious behavior is universal in LND. All information regarding the housekeeping hypoxanthine phosphoribosyltransferase 1 (*HPRT1*) gene that encodes the soluble cytoplasmic (HGprt) enzyme as well as the pathological conditions associated with the deficiency of HGprt activity found in LND and its variants: Lesch-Nyhan variants, LNVs, due to a partial deficiency of HGprt enzyme activity are described in [1]–[7]. These LNVs patients are characterized by consequences of overproduction of uric acid and variable spectrum of neurological manifestations, without the self-injurious behaviors [1]–[7]. How the loss of HGprt enzyme function affects the brain to cause the neurobehavioral syndrome in LND/LNVs, especially the self-injury of LND? For such a question, up to present, there is no valid answer. This has made difficult for the treatment development and has led to the absence of effective LND treatments [1]–[7]. Recently, however, it was demonstrated, for the first time, that expression of the β-amyloid precursor protein (APP) gene is under epigenetic regulation resulting in the presence of several APP messenger (APP-mRNA) isoforms encoding diver APP protein isoforms ranging from 120 to 770 amino acids (with or without mutations and/or deletions), and APP-mRNA isoforms with a deletion followed by an insertion (INDELS) accounted for epigenetic mechanisms in the regulation of alternative APP pre-mRNA splicing due to epigenetic modifications and/or epistasis (gene-gene interactions) as well as to epigenetic control of genomic rearrangements of *APP* gene [8],[9]. In addition, a report on the quantification of various APP-mRNA isoforms in biological samples, especially for identifying the most abundant one that may be decisive for the normal status or disease risk has been described and applied for identifying the defective APP-mRNA isoform in LND. The results indicated, for the first time, a role for epistasis between mutated *HPRT1* and *APP* genes affecting the regulation of alternative APP pre-mRNA splicing in which APP-mRNA isoform of 624 bp, with a deletion starting after 49 bp of the 5′ end of exon 3 followed by a complete deletion of exons 4–15, mutations in exon 1: c.22C > T, p.L8F, and exon 3: c.269A > G, p.Q90R encoding APP_207_ isoform was the most abundant one in most of the LND patients. This APP_207_ isoform would be responsible for the neurobehavioral syndrome observed in these patients [10]. Furthermore, there were also some reported cases of LND/LNVs developing thrombosis [11]–[13] while APP is an important regulator of vein thrombosis and controls coagulation and neutrophil extracellular traps (NETs) formation via the Kunitz-type serine protease inhibitor (KPI)-containing the α soluble fragment of APP (APPsα fragment) that were demonstrated in vitro to be effective inhibitors of the coagulation FXa, FIXa, FXIa, and FVIIa:tissue factor complex [14]. Then, APP pathway could be implicated in the development of neurological features as well as thrombotic events of LND/LNVs. Otherwise, the surface expression of HGprt enzyme was also observed in several somatic tissue cancers [15]–[21] while an important function of APP as a tumor growth factor in the pathogenesis of several somatic tissue cancers has been suggested and APP as well as APP-like protein-2 (APLP2) are deregulated in cancer cells and linked to increased tumor cell proliferation, migration, and invasion [22],[23]. These findings suggest an emerging role of HGprt in cancer development. The present review provides a discussion about these findings and suggests a potential molecular link between APP and HGprt via epistasis between *HPRT1* and *APP* genes affecting the regulation of alternative APP pre-mRNA splicing. As a perspective, expression vectors for HGprt enzyme and APP are constructed [24] and they could be used as tools for clarification of these issues.

## 2. Cases reports

### 2.1. HGprt and thrombosis from LND and LNV patients

Immamura et al. [11] reported the association between LND and hypercoagulability in 4 patients with elevated plasma fibrinopeptide A (FPA) level in which one patient complicated with cerebral infarction. Riaz et al. [12] reported extensive hypercoagulability in a patient with Leshh-Nyhan variant (LNV) manifesting as recurrent myocardial infarctions, thromboembolic disease, and thrombus formation in bronchi despite triple anti-thrombotic therapy. Tewari et al. [13] reported a LND patient manifesting cerebral venous thrombosis and right cortical venous thrombosis (frontal and parietal veins) with thrombosis of the superior and left transverse sinus veins and a venous infarct in the right superior frontal gyrus (with cortical laminar necrosis). Cortical venous congestion was noted in the rest of the frontal and parietal cortices on the right.

### 2.2. HGprt and cancer

Townsend et al. [15] reported the surface expression of HGprt enzyme on the plasma membrane of certain cancer cell lines such as the human non-small lung cancer cell lines NCI-H460 and A549. Recent works has showed that not only is the enzyme upregulated within malignant tumors such as lung, breast, colon, endometrial, and prostate cancers, it also has significant surface localization within some cancer cells such as colorectal cancer cell lines HT29, SW620, and SW480 [16]–[21]. An emerging role of HGprt as a biomarker not only for the characterization of human cancer, but also for its potential treatment, was therefore reported [18]–[21].

## 3. Discussion

A major unsolved question is how the loss of HGprt enzyme function affects the brain to cause the neurobehavioral syndrome in LND/LNVs. Histopathological studies of autopsy tissues from LND patients revealed no signs suggestive of a degenerative process in any brain region [25]. On the other hand, and at the biochemical level, there was strong evidence that the neurological impairments in LND/LNVs were due to the effect of HGprt deficiency on the neural development, mainly, but not only, related to dopaminergic pathways [26],[27]. Nevertheless, none of these studies showed the pathogenic mechanism whereby HGprt deficiency affects the neuronal development, and the mechanism by which features of LND/LNVs result from impaired purine metabolism is still not well understood. However, it was also documented that:

- adhesion of HGprt-deficient neuroblastomas as well fibroblasts from patients with LND/LNVs exhibited dramatically enhanced adhesion compared to control [28], and could have consequences for the maturation of the central nervous system, as seen in the small brain size of LND/LNVs children [29]–[31];

- Alzheimer's disease (AD) shares gene expression aberrations with purinergic dysregulation of HGprt deficiency [32];

- role for the APP is a key developmental gene related to cell-cell or cell-substrate adhesion, generation of neurons, their differentiation and migration, neurite outgrowth, regulation of synaptic function, and is important for brain morphology and highly coordinated brain function such as memory and learning has been suggested [33],[34].

In an attempt to search for a link between LND/LNVs and APP, examination of the APP-mRNA profile, the genomic APP-DNA from the fibroblast of normal subjects and LND/LNVs patients was then performed. The results obtained showed, for the first time, that different isoforms of APP-mRNA can exist [8],[9] and the most abundant one quantitatively may be decisive for the normal status or disease risk in which the APP_207_ isoform was found in most of the LND patients [10]. Hence, the APP pathway is possibly implicated in the development of neurological features of LND/LNVs.

### 3.1. APP-HGprt and thrombosis in LND/LNVs

The findings as described in [11]–[13] support the impact of APP on LND/LNVs and suggest a potential molecular link between APP and HGprt enzyme via epistasis between mutated *HPRT1* and *APP* genes. Indeed, (1) the KPI domain, which is located in exon 7: amino acid residues 291–341 of the extracellular domain of APP_770_ and APP_751_ isoforms [34],[35], has been shown *in vitro* to be a potent inhibitor of the coagulation FXa, FIXa, FXIa, and FVIIa: tissue factor complex [14],[36],[37]. Furthermore, in addition to brain, APP is also expressed in extraneuronal tissues, mostly in platelets in which the APP_751_ and APP_770_ isoforms are expressed [14]. On platelet activation and under physiologic conditions, the majority of APP is processed via the non-amyloidogenic pathway via α-secretase, which is activated by a Ca^2+^/calmodulin-dependent mechanism [14],[34],[35]. Then, the processing of APP from platelets releases the KPI-containing soluble APPsα fragments [34],[35] that are analogous to protease nexin-2 (PN-2) [14],[36],[37]. PN-2/APP and its KPI domain have been demonstrated *in vitro* to be potent inhibitors of trypsin, chymotrypsin, epidermal growth factor binding protein, the γ subunit of nerve growth factor, and several key prothrombotic proteinases including factor XIa, factor IXa, factor Xa, and tissue factor: factor VIIa complex [14],[36],[37]. *In vivo* studies on APP/KPI^R13I^ mutant mice (mutation of the center basic arginine 13 residue of the KPI domain to the similar sized, but hydrophobic, isoleucine) [37], and APP-knock out mice have indicated that, as a result, APP negatively controls thrombosis [14]. It is important to note herein that (a) APP and APLP2 (possesses a highly conserved KPI domain that is highly homologous with the one contained in APP) are expressed ubiquitously throughout the body, mostly abundant in the nervous system; whereas APP-like protein-1 (APLP1) does not contain a KPI domain, is predominantly expressed in the nervous system [34],[35]. Here, similar to APP, APLP2 has been also shown *in vitro* and *in vivo* to have inhibitory activity against these hemostatic enzymes factors and regulates thrombosis [36]. These findings demonstrate an important role for platelet APP and APLP2 (expressed at a lower level) are both proteolytic inhibitors, through the KPI activity of the protein, that possess overlapping and shared activities contributing to the regulation of blood clot formation, limiting thromboembolic diseases as well as cerebral venous thrombosis [14],[36],[37]; (b) the severity of the prothrombotic risk of APP and, more recently, APLP2 have been proposed as cerebral anticoagulants [36]; (2) as previously mentioned, expression of APP gene is under epigenetic regulation resulting in the presence of several APP-mRNA isoforms (with or without mutations and/or deletions), encoding diver APP protein isoforms accounted for epigenetic mechanisms in the regulation of alternative APP pre-mRNA splicing was reported [8],[9]. In addition, a report on the quantification of various APP-mRNA isoforms in biological samples, especially for identifying the most abundant one that may be decisive for the normal status or disease risk has been described and applied for identifying the defective APP-mRNA isoform in LND [10].

Taking into consideration these findings, a miss regulation of alternative APP pre-mRNA splicing could lead to the presence of the most abundant APP-mRNA isoform that would be a defective one encoding consequently a defective APP protein isoform (or its proteolytic fragments) with mutation and/or deletion in the KPI domain such as the APP/KPI^R13I^ [37] or the 624 bp of APP-mRNA isoform encoding APP_207_ isoform as described in [10]. These defective isoforms could affect anticoagulant functions and abolishes therefore their anti-thrombotic activity. In this case, the overlapping compensatory effect of APLP2 would be decisive for the preservation of anti-thrombotic activity. This could explain the development of thrombosis from some LND/LNVs patients as described in [11]–[13].

### 3.2. APP-HGprt and cancer

Salvage enzyme, such as HGprt and is known as a housekeeping protein, in which its important role is responsible for the production of nucleotides such as GTP and ATP that are necessary to providing energy for several cellular process as well as for regulating cell proliferation [1],[2]. Expression of *HPRT1* is cytosolic within all normal cells and maintained stable and low levels in normal tissue. Here, some questions remain to be elucidated such as (a) How HGprt is able to localize to the surface?; (b) Does it provide any functional advantage to the cancer cell?; (c) Determine the reason some cells that express HGprt on the surface while others do not? The observed surface expression of HGprt on certain malignancies makes it promising as a biomarker in the early diagnosis of cancer such as lung and colorectal cancer [15],[16],[18]. Surface expression of colorectal cancer cells has been also observed for the vitamin D3 receptor, and serves as a maker for such a cancer [18]. It is important to note that APP is ubiquitously expressed in a broad spectrum of cell types including non-neuronal cells, and it is suggested to be involved in the growth of these cells [34],[35],[38], while the nature of APP has been mainly studied in neuronal cells due to its pathological significance in AD. Recently, increasing evidence suggests an important function of APP as a potent tumor growth factor in the pathogenesis of several somatic tissue cancers, and APP as well as APLP2 are deregulated in cancer cells and linked to increased tumor cell proliferation, migration, and invasion [22],[23]. These findings suggest a potential link between APP and HGprt in cancer development. Indeed, it was demonstrated that expression of *APP* gene is under epigenetic regulation and a role for epistasis between *HPRT1* and *APP* genes affecting the regulation of alternative APP pre-mRNA splicing was also suggested [8],[9],[10]. A miss regulation could lead to the presence of the most abundant APP-mRNA isoform that would be a defective one encoding consequently a defective APP protein isoform (or its proteolytic fragments) capable of promoting cancer growth. In cancer, cells rapidly divide, the need for nucleotides increases, and as a result HGprt is upregulated and some cancer cells express HGprt on the surface for the purpose of inducing changes in the metabolism and activity to maintain rapid tumor cell proliferation.

## 4. Conclusion and perspective

Epistasis is important, ubiquitous, and has become a hot topic in complex disease genetics such as AD, schizophrenia, autism, cancer, etc. in recent years. A gene does not function in isolation and by itself, but rather acts with other genes in a network, to influence complex traits of the complex disorders. However, the data supporting epistasis in complex human diseases are emerging slowly. This is due to different difficulties that we face in detecting and characterizing epistasis, such as challenges of modeling non-linear interactions, and in the interpretation of results [10],[39]–[41]. APP, a housekeeping gene and an endogenous ligand (http://www.genenames.org/genefamilies/ENDOLIG) [10],[34],[35], is an important molecular hub at the center of interacting pathways and acts as a permissive factor for various cellular functions, and therefore it is not surprising that altered APP processing may affect neuronal as well as non-neuronal cellular functions through a host of altered cellular and molecular events found in human diseases. Furthermore, α-, β- and γ-secretase processing of APP (at the N-and C-terminals of the Aβ sequence) also occur under physiological conditions; this indicates that all fragments of APP, including the Aβ peptide, are part of normal physiology. The targeting of the components of APP processing as a pharmacologic strategy will not be without consequences. Therefore, it is important to continue to investigate the normal function of APP. Understanding its physiological function will not only provide insights into the pathogenesis of diseases but may also prove vital in the development of an effective therapy. The role of epigenetics in rare diseases is a key issue in molecular physiology and medicine because the understanding about the mechanisms that explain the influences of epigenetic regulation in rare diseases will provide useful principles for other common and complex disorders such as AD. Epigenetic regulation determines not only what parts of the genome are expressed but also how they are spliced [23].

In conclusion, the examples discussed here suggest strongly a potential molecular link between APP and HGprt via epistasis between *HPRT1* and *APP* genes, and highlight the impact of alternative splicing (AS) process on human disease, and clearly show that how AS is dynamically regulated and generates isoform diversity with critical functions and a misregulation of AS plays a large role in numerous human diseases. An accurate quantification of various APP-mRNA isoforms from different tissues for identification the most abundant APP-mRNA isoform that may be decisive for the normal status or disease risk is needed and antisense drugs are the potential treatments [10],[42].

As a perspective, for clarification of these issues, it is necessary to study the HGprt enzyme and APP using expression vectors for exploring their impacts on LND as well as other human diseases, especially the ones related to APP such as AD and cancer [23]. For such a purpose, the construction of expression vectors for HGprt and APP was performed [24]. These expression vectors, with or without the glycosyl-phosphatidylinositol (GPI) anchor, could be used as tools for (a) studying the effects of mutation on HGprt enzyme found from different LND/LNVs patients; (b) studying the emerging role of *HPRT1* gene in cancer, especially exploring the effects for the surface expression of *HPRT1* gene; (c) exploring the mechanism linking HGprt deficiency, purinergic pathways, and neural dysfunction of LND; (d) exploring the structure and the physiologic function of APP; (e) studying intermolecular interactions between APP and HGprt enzyme.

It is also important to note herein that these expression vectors, especially the one with GPI anchor can be used as a model for the construction of expression vectors for any protein targeting to the cell plasma membrane for studying intermolecular interactions and could be therefore useful in the vaccines as well as antiviral drugs development (studying intermolecular interactions between the spike glycoprotein of the severe acute respiratory syndrome coronavirus 2, SARS-CoV-2, as well as its variants and the angiotensin-converting enzyme 2, ACE2, in coronavirus disease 2019 (COVID-19) [43],[44], for example).
